# Connectedness as a candidate pathway linking psilocybin retreat experiences to sustained wellbeing: a prospective naturalistic study

**DOI:** 10.3389/fpsyt.2026.1924069

**Published:** 2026-10-28

**Authors:** Maria Åkesson, Pehr Granqvist, Robert Johansson, Joel Gruneau Brulin

**Affiliations:** Department of Psychology, Stockholm University, Stockholm, Sweden

**Keywords:** connectedness, mystical experiences, psilocybin, psychedelics, retreat, wellbeing

## Abstract

**Background:**

Psychedelic retreats are increasingly used to promote wellbeing outside clinical settings, yet the psychological processes associated with sustained improvements in wellbeing remain poorly understood. From a public mental health perspective, identifying mechanisms that support positive mental health beyond symptom reduction is of growing importance.

**Methods:**

This naturalistic longitudinal study followed 173 participants attending guided psilocybin retreats conducted within a structured framework of preparation, dosing, and integration. Mental wellbeing was reported before the retreat and across a 12-month follow-up period. Longitudinal changes were examined using linear mixed-effects models. Mediation analyses investigated whether changes in connectedness, neuroticism (anxiety facet), spiritual transcendence, and absorption were associated with changes in wellbeing. Mystical-type experiences were examined as potential predictors of these psychological changes.

**Results:**

Participants reported substantial and sustained improvements in mental wellbeing over 12 months, moving from levels below Swedish population norms at baseline toward comparable levels at follow-up. Connectedness showed the strongest statistical association with improvements in wellbeing and accounted for the largest proportion of variance in wellbeing change, followed by reductions in the anxiety facet of neuroticism and absorption. Spiritual transcendence was associated with wellbeing improvements when examined separately but did not retain unique effects in the multiple mediation model. More intense mystical-type experiences predicted greater increases in connectedness, spirituality, and absorption, as well as greater reductions in neuroticism (trait anxiety). The association between mystical experiences and wellbeing was substantially attenuated when connectedness was included in the model, suggesting that connectedness may act as a candidate mediator linking mystical-type experiences to longer-term wellbeing, though the observational design precludes causal inference.

**Conclusions:**

These findings extend prior work on psychedelic-assisted therapy to naturalistic retreat settings, suggesting that connectedness may represent a key psychological process linking psychedelic retreat experiences to sustained improvements in mental wellbeing. From a public mental health perspective, the results highlight the potential importance of social and relational factors in promoting wellbeing beyond the treatment of mental illness. Future controlled studies are needed to clarify the relative contributions of psychedelic, social, and contextual components.

## 1 Introduction

### 1.1 Mental wellbeing

Mental health is increasingly recognized as a population-level concern rather than solely an individual clinical one. From a public mental health perspective, promoting wellbeing and preventing mental ill-health across the population are considered central public health goals, extending beyond the treatment of diagnosed mental disorders (1). Among the most consequential population-level risks is social disconnection. A joint statement by the United States, Japan, Morocco, Sweden, Kenya and Chile at the WHO Executive Board highlighted the importance of social connection to health and well-being, noting that loneliness and social isolation are clearly linked to outcomes such as cardiovascular disease, diabetes, depression and cognitive decline, and carry a risk of early death comparable to that of smoking and excessive drinking (2). Loneliness remains an independent risk factor for depressive symptoms even after adjustment for demographic and psychosocial variables (3), and disconnection from others and the environment is broadly linked to poorer physical and mental health, whereas social connectedness supports wellbeing and recovery (4). Identifying experiences that foster connectedness and sustained wellbeing, and the settings in which they occur, is therefore a pressing public mental health priority.

Mental wellbeing, as a core component of health, cannot be reduced to the absence of illness. It encompasses subjective wellbeing and functional capacity — the ability to cope with challenges and sustain meaningful relationships (5, 6). Contemporary frameworks describe wellbeing as feeling good and functioning well across hedonic, eudaimonic, and relational dimensions (7), reflecting positive psychological functioning such as optimism, purpose, and social connectedness rather than merely the absence of mental health problems. Mental wellbeing is shaped not only by individual factors but also by social, environmental, and structural determinants (1). A public mental health perspective therefore extends beyond questions of individual change to consider wellbeing at the population level. It also encourages comparison with population benchmarks, situating the wellbeing of a given sample within a broader societal context and enabling interpretation relative to the wider population.

### 1.2 Psychedelics and mental wellbeing

A rapidly expanding evidence base links psychedelics, particularly psilocybin, to improvements in mental health. Systematic reviews, meta-analyses, and randomized controlled trials (RCT) report significant reductions in depressive symptoms across a range of clinical populations, including major depressive disorder and cancer-related distress (8–12), although small samples and difficulties maintaining blinding continue to limit the strength of causal conclusions (13).

Alongside RCTs, a growing body of naturalistic research examines psychedelics in real-world contexts. While the tightly controlled conditions of RCTs support causal inference (8, 10–12), they can limit ecological validity. Naturalistic studies complement RCTs (14) by showing how interventions function under typical conditions, yielding real-world evidence that can support inferences about therapeutic effects and mechanisms. Such studies report reductions in anxiety, neuroticism, and depression, increases in mindfulness and openness, and enhanced social connectedness during and following psychedelic group ceremonies (15–17), though variability in settings and reliance on self-reports constrain reproducibility and challenge validity.

Importantly, psychedelic experiences appear to influence psychological processes that are closely related to wellbeing. Emerging evidence suggests that improvements in mental health may be accompanied by broader changes in how individuals relate to themselves, others, and the world around them, as well as by shifts in personality-related and experiential factors (8, 16, 18). Several of these psychological processes, together with acute psychedelic experiences, have been proposed as potential contributors to longer-term improvements in wellbeing. Acute psychedelic experiences encompass a range of subjective phenomena. These include mystical-type experiences, characterized by experiences of unity or interconnectedness, transcendence of ordinary time and space, ineffability, and a sense of sacredness or deeply felt positive mood (19, 20). Other experiential phenomena, such as emotional breakthrough and psychological insight, have also been investigated in relation to psychological outcomes (21), and broadly involve the emergence and resolution of previously difficult-to-access emotional material (22).

However, these effects do not occur in isolation from context. Accumulating evidence indicates that the setting of psilocybin administration, including the quality of psychological support, plays a central role in shaping both acute experiences and long-term outcomes. A supportive therapeutic relationship has in clinical studies been associated with key experiential features such as emotional breakthrough and insight, as well as clinical improvement (21). A systematic review of 22 trials found substantial convergence in delivery, including structured preparation, guided support during sessions, post-session integration, and carefully controlled environmental conditions (23). Notably, uncontrolled settings further increase the risk of adverse outcomes (24). Individuals higher in neuroticism tend to experience more challenging sessions and may find it harder to “let go” and reduce negative reactivity, underscoring the importance of skilled psychological support throughout the process (24).

In the present prospective naturalistic study, we focus on four presumed mediators: connectedness, neuroticism (trait anxiety), absorption, and spirituality, together with mystical experiences to test if they can help explain how psychedelic practices are followed by improvements in mental wellbeing.

### 1.3 Connectedness and other psychological contributors to wellbeing

Among the modifiable contributors to wellbeing, connectedness may be especially central. Beyond the documented harms of loneliness, positive forms of connection appear to actively support flourishing; nature connectedness, for example, is associated with higher wellbeing (25). More broadly, feeling connected to oneself, others, and the wider world has been proposed as an important contributor to psychological wellbeing and flourishing, and as a potential mechanism underlying positive psychedelic outcomes (18). Notably psilocybin experiences have been associated with enhanced feelings of connection to the self, others, nature, and the wider world (16, 18). Qualitative reports have further identified increased connectedness as a central feature of perceived psychological improvements following psilocybin experiences (26). Rather than representing one factor among many, connectedness may function as a central integrative process through which individuals experience meaning, belonging, and psychological coherence (27). Its documented association with wellbeing, together with evidence that psychedelic experiences can enhance feelings of connection, suggest that connectedness may represent a key pathway linking acute psychedelic experiences to longer-term improvements in wellbeing (26).

Spirituality, conceptualized here as the capacity to experience meaning, purpose, and a broader perspective on life that extends beyond immediate circumstances (28), may support coping, emotional regulation, and overall wellbeing, consistent with evidence linking spiritual practices to greater psychological wellbeing (29, 30). Also, neuroticism is a robust risk factor for psychopathology, particularly depression and anxiety (31, 32). Although relatively stable, neuroticism can shift following major life experiences (33). Psychedelic experiences have been associated with reductions in neuroticism, suggesting the potential for lasting personality change (8, 15, 17). Absorption, the disposition to become deeply engaged in sensory and imaginative experience (34), may also serve an important role through its link to openness, altered states of consciousness, and positive affect (35, 36). However, higher levels of absorption may also indicate dissociative tendencies (37, 38), suggesting both potential benefits and vulnerabilities.

### 1.4 Mystical-type experiences as a predictor of sustained wellbeing

The intensity and character of the subjective experiences during the acute psychedelic state appear to be important for understanding subsequent therapeutic and psychological effects (39, 40). Among these experiences, mystical-type experiences have received particular attention. They are typically characterized by a sense of unity or interconnectedness, transcendence of time and space, ineffability, and deeply felt positive mood (19, 20). Experimental studies show that psilocybin can reliably occasion mystical-type experiences, which participants frequently rate among the most personally meaningful experiences of their lives (41). Moreover, the intensity of such experiences has been associated with enduring changes in attitudes, behavior, and psychological functioning, and has been proposed as an important contributor to long-term wellbeing following psychedelic experiences (10, 20, 40, 42). Consistent with this, a review of 44 studies found that the majority of reported positive mental health outcomes were associated with a higher degree of psychedelically facilitated mystical-type experiences (20). Notably, this association appears substantially weaker in naturalistic settings than in protocol-based clinical trials (39), highlighting the need to examine it in retreat contexts specifically. Conceivably, mystical-type experiences may be associated with subsequent psychological improvements by enabling the more durable shifts in connectedness and related processes described above. Their role in predicting long-term wellbeing in naturalistic retreat settings, however, remains insufficiently understood.

### 1.5 The present study

This naturalistic longitudinal study addresses several of the aforementioned gaps by examining real-world outcomes of psilocybin retreats on mental wellbeing over 12 months. Unlike controlled clinical trials, we studied a retreat setting in which all participants received psychologist-guided group preparation and integration — an inherently social environment involving shared experience, collective meaning-making, and opportunities for interpersonal connection. Such settings may serve not merely as a context for psychedelic administration but as an active component shaping psychological outcomes, supporting the integration of acute experiences into everyday life. With a relatively large sample attending a single standardized retreat model at different times, the study enhances ecological validity and may contribute to the limited literature on long-term wellbeing outcomes.

### 1.6 Aim and hypotheses

The aim of this study was to investigate changes in mental wellbeing from before to after a psychedelic retreat, and over a 12-month follow-up period. The aim was also to examine the role of potential mediators — connectedness, spiritual transcendence, neuroticism (anxiety facet), and absorption — in the observed changes in wellbeing, and to investigate whether the intensity of mystical experiences during the retreat predicted changes in wellbeing and in the proposed mediators. The study was part of a larger longitudinal project, see 43. The present study addressed the following research questions:

Primary Research Question

RQ1: Does mental wellbeing change favorably among individuals who attend a psilocybin retreat, from pre to post retreat and over a 12-month period?

Hypothesis 1: Mental wellbeing would increase following participation in the retreat and remain higher than baseline during follow-up.

Secondary Research Questions

RQ2: Do psychological changes — in connectedness, spiritual transcendence, neuroticism (anxiety facet), and absorption — mediate the presumed improvements in mental wellbeing, jointly or in isolation?

RQ3: Do mystical-type experiences during psychedelic intake predict improvements in wellbeing and changes in the proposed mediators, and is the association between mystical-type experiences and wellbeing mediated by changes in the presumed mediators?

Hypothesis 2: Greater intensity of mystical-type experiences during the retreat would predict greater increases in wellbeing at follow-up.

Both hypotheses were preregistered (43); all other analyses were exploratory. In addition, a supplementary analysis compared participants’ wellbeing with Swedish population benchmarks.

## 2 Materials and method

### 2.1 Study design

The present study forms part of a preregistered naturalistic longitudinal project conducted at Stockholm University (43). The study employed a within-subject repeated-measures design to examine changes in mental wellbeing from pre- to post-retreat and across a 12-month follow-up period. In addition, the study investigated whether psychological variables (connectedness, spiritual transcendence, neuroticism (anxiety facet), absorption, and mystical-type experiences) were associated with changes in wellbeing.

All individuals who enrolled in psilocybin retreats organized by a particular company (“Nysnö”) between 2022 and 2024 were invited to participate. Participants provided informed consent and completed seven online surveys administered before, during, and after the retreat.

The protocol was submitted to the Swedish Ethical Review Authority before study initiation (see further details in 4.3 Ethical and Legal Considerations) collected from participants attending one of the retreats

### 2.2 Participants

A total of 207 adults enrolled in the study. Of these, 173 participants met the inclusion criteria for the main longitudinal analyses by completing the baseline assessment (T1) and at least one follow-up assessment between T3 and T7. Data collection was prematurely halted due to the closure of the web-based platform used for data gathering in late 2024, resulting in incomplete follow-up data for some participants (see **Table 1**). Of the participants included, around half were women (n = 97; 56.1%), and the median age was 49 years (range 27–81). Just over half of the sample (n = 98; 56.6%) reported having no previous experience with psychedelic substances.

**Table 1:** Participants and demographic characteristics across data collection time points, including the total retreat sample.

Time point	*n* (% of T1)	Mean age (range)	Female *n* (%)	Psychedelic-naïve *n* (%)
T1: two weeks before	173	49.9 (27–81)	97 (56.1%)	98 (56.6%)
T2: one day before	154 (89.0%)	50.6 (27–81)	85 (55.2%)	91 (59.1%)
T3: one day after	158 (91.3%)	50.1 (27–81)	90 (57.0%)	96 (60.8%)
T4: two weeks after	152 (87.9%)	49.6 (27–81)	85 (55.9%)	87 (57.2%)
T5: three months after	104 (60.1%)	49.0 (27–72)	61 (58.7%)	62 (59.6%)
T6: six months after	75 (43.4%)	49.8 (27–71)	42 (56.0%)	45 (60.0%)
T7: twelve months after	56 (32.4%)	49.1 (31–65)	29 (51.8%)	28 (50.0%)

Time points are given relative to the retreat.

Eligibility for attending the retreat required being at least 18 years old and free from psychiatric or medical conditions associated with elevated risk for psilocybin administration (e.g., bipolar disorder, family history of psychosis, previous cardiac events, hemorrhagic stroke, uncontrolled hypertension). All applicants completed a health declaration and underwent medical screening by a licensed physician. Individuals were required to abstain from psychiatric medications that could interact with psilocybin.

Participants paid a program fee of approximately SEK 27,000 excluding VAT (approximately USD 2,600) program which covered accommodation, program content, psychologist-led preparation, support during the retreat, and integration sessions. The program emphasized personal development and self-exploration rather than treatment of mental health difficulties.

### 2.3 Intervention

The Nysnö retreat program was developed and facilitated by licensed Swedish psychologists and conducted in the Netherlands, where psilocybin truffles are legal to ingest. The data in the current study was collected from participants attending one of the retreats held between 2022 and 2024. The four-week program comprised three phases: *Preparation, Retreat*, and *Integration* (See **Figure 1**). Across the three phases of the program, facilitators played an active role. At least one licensed psychologist was included in the facilitation team for each retreat. The facilitation team was responsible for planning all components of the program and a licensed psychologist conducted primary screening, accompanied by a medical doctor, for all participants, as well as any additional in-depth screening prior to participation. The program included a total of four individual sessions with a facilitator per participant – one session during the preparation phase, two sessions during the retreat, and one session during the integration phase. In addition to individual sessions, facilitators led multiple group meetings across phases, including two group sessions during preparation, approximately five during the retreat, and two during the integration. During the preparation and retreat phases, facilitators led structured group exercises and workshops aimed at fostering psychological safety, trust, group cohesion, mindfulness, and intention-setting to support vulnerable engagement in the process. In the integration phase, facilitators guided structured group discussions and reflective exercises to support meaning-making, revisit participants’ initial intentions, and assist in translating experiences from the retreat into everyday life. Across all phases, the facilitators’ role was supportive and facilitative, focusing on psychological safety, reflection, and integration, rather than delivering a formal psychotherapeutic intervention. For a full description of the retreat program see Bromberg, Eriksson, and Råback (44).

**Figure 1:**
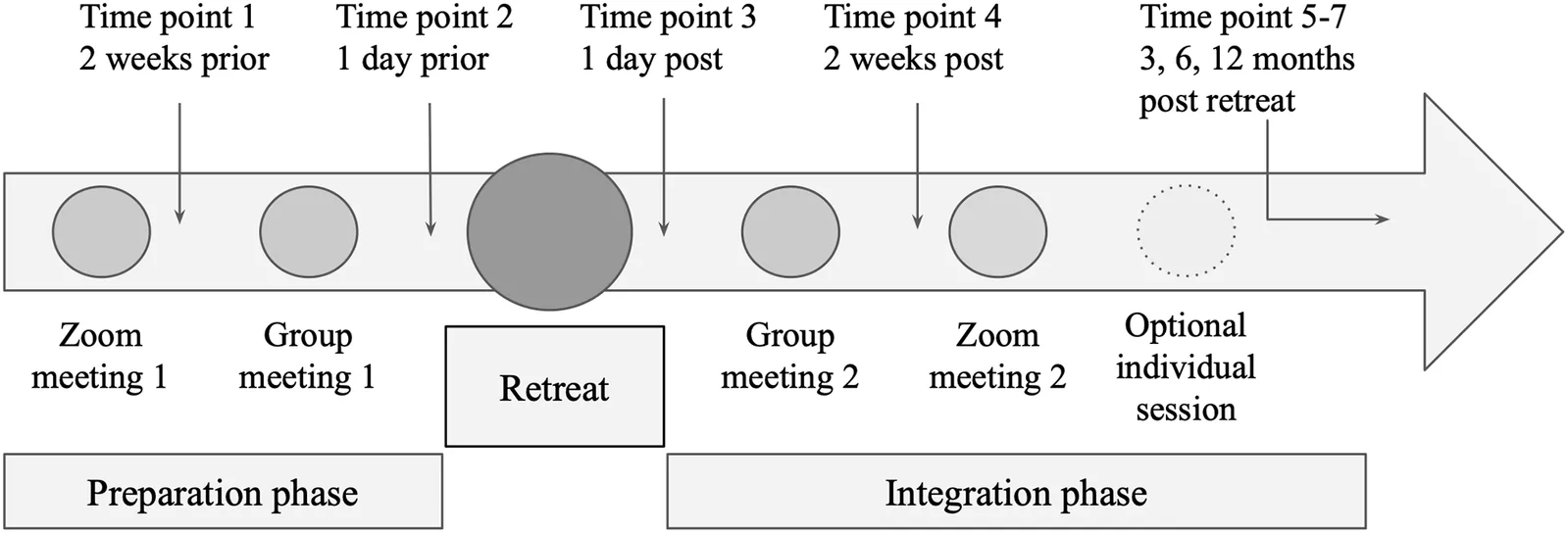
Flowchart of phases, group meetings, and survey administrations across time points (T1–T7). The image depicts group-based activities (below arrow) and survey administrations (above arrow) across phases. In addition to the group sessions shown, participants also engaged in individual psychologist-led sessions during the preparation, retreat, and integration phases, not displayed in the figure.

#### 2.3.1 Preparation phase

Participants completed an initial screening to evaluate potential risks and ensure the program’s suitability based on participants’ individual life contexts, and one individual preparatory session with a psychologist. Two group sessions (face to face or via zoom) were held to establish psychological safety, trust, and group cohesion through structured exercises, mindfulness practices, and intention-setting.

#### 2.3.2 Retreat phase

The retreat took place over four days in a Dutch rural setting and included up to 20 participants per group. Three facilitators (at least one a licensed psychologist) guided the process. Participants completed two group-based psilocybin sessions. Psilocybin was administered orally in the form of fresh psilocybin-containing truffles (Psilocybe Hollandia) on day 1 and day 3 of the retreat. Dosing sessions were conducted with participants lying on mattresses arranged in a circle and facilitators providing continuous emotional and psychological support and ensuring participant safety throughout the sessions. The first psilocybin session typically involved a lower yet moderate to strong introductory dose (*M* = 20.68 g, *SD* = 2.72, *Mdn* = 20 g, range = 15–30 g) while the second session typically involved a higher dose (*M* = 55.57 g, *SD* = 20.08, *Mdn* = 50 g, range = 10–150 g) adjusted based on the participant’s first-day experience. Participants received a single initial dose during each session, with the option of an additional supplemental dose later in the session, reflected in the wider dose range observed during the second administration. Higher doses were distributed across approximately three hours to account for tolerance development and to maintain psychological safety. Doses reflect wet weight and were self-reported retrospectively by participants. Psilocybin content was not analyzed, but has been reported at approximately 0.6–1.7 mg per gram of fresh truffles (45), corresponding to roughly 12–34 mg and 30–84 mg of psilocybin for the median doses in the first and second sessions, respectively. Participants had access to both indoor and outdoor areas, and live or recorded music curated by the facilitators accompanied the sessions. Participants were encouraged to direct attention inward while remaining respectful of others’ experiences.

#### 2.3.3 Integration phase

Following the retreat, participants attended two structured group integration sessions and one individual session, aimed at facilitating reflection on the experiences, re-establishing connections with the group, and transitioning insights gained during the retreat into everyday life. These sessions also emphasized revisiting participants’ original intentions as a means of anchoring their experiences. Participants were further encouraged to engage in follow-up activities such as integration groups and breathwork, maintain contact with one another beyond the organized sessions, and could optionally book an additional one-on-one integration session.

### 2.4 Measurements

Demographic data were gathered at T1, two weeks prior to the retreat, and included participants’ age, gender, and prior psychedelic experiences. All participants could choose to answer the survey.

#### 2.4.1 Mental wellbeing

Mental wellbeing was assessed using the 7-item Short Warwick-Edinburgh Mental Wellbeing Scale (SWEMWBS) (46, 47) at all seven time points (T1–T7). The scale uses a 5-point Likert scale ranging from 1 (“None of the time”) to 5 (“All of the time”), with higher scores indicating greater wellbeing. Example item: “I’ve been feeling optimistic about the future”. SWEMWBS focuses on positive aspects of mental health and has demonstrated strong psychometric properties, with Cronbach’s alpha values typically ranging from .80 to .85 (48, 49). The scale has shown consistent reliability and validity across different populations, including Scandinavian samples. Cronbach’s alpha in the present study ranged from .87 at T1 to .92 at T4.

#### 2.4.2 Connectedness

Connectedness was measured using the 19-item Watts Connectedness Scale (WCS) (18) at T1 and T4–T7. The scale assesses connectedness across the domains Self, Others, and the World, which can also be combined into a general connectedness factor. Items are rated on a 7-point Likert scale ranging from 1 (“Strongly disagree”) to 7 (“Strongly agree”), with higher scores indicating greater connectedness. Example item: “My head has been connected with my heart/my feelings”. The WCS has demonstrated strong psychometric properties, including good internal consistency and construct validity, with Cronbach’s alpha values above .80 for the subscales (18). In the present study the combined scale was used, displaying Cronbach’s alphas ranging from .91 at T1 to .95 at T5. Connectedness was not administered for the first timepoints for the first 30 participants, as this measurement was added two months into the data collection.

#### 2.4.3 Spiritual transcendence

The Spiritual Transcendence Scale (STS), including only the connectedness and universality subscales (28) was administered at T1 and T4–T7. These 16 items assess spiritual transcendence, defined as the ability to perceive life from a broader perspective beyond immediate time and place. Items are rated on a 5-point Likert scale ranging from 1 (“Strongly disagree”) to 5 (“Strongly agree”), with higher scores indicating greater spiritual transcendence. Example item: “I still have strong emotional ties with someone who has died”. The STS has demonstrated strong psychometric properties, with Cronbach’s alpha values typically ranging from .80 to .90, indicating high internal consistency (28). In the present study, one general factor of spiritual transcendence was used, demonstrating Cronbach’s alphas ranging from .93 at T1 to .95 at T7.

#### 2.4.4 Neuroticism – anxiety facet

Trait anxiety was measured using the 4-item IPIP-NEO Anxiety Facet (50) at T1 and T4–T7. The scale assesses the anxiety facet of neuroticism (e.g. “I worry about things”), with items rated on a 5-point Likert scale ranging from 1 (“Strongly disagree”) to 5 (“Strongly agree”). Higher scores indicate greater trait anxiety. The measure has demonstrated strong convergent and criterion validity, with reported Cronbach’s alpha values around .80 (50). In the present study Cronbach’s alpha ranged from .84 at T4 to .93 at T7.

#### 2.4.5 Absorption

Absorption was assessed using a short 12-item version of Tellegen Absorption Scale (51; original scale: 34) at T1 and T4–T7. The scale measures the disposition for episodes of complete attention and openness to immersive, self-altering experiences (e.g. “Sometimes it happens that I go completely into nature or art and that I feel as if my whole state of consciousness is somehow temporarily changing”). Items are rated on a 5-point Likert scale ranging from 1 (“Never”) to 5 (“Always”), with higher scores indicating greater absorption. The original 34 item scale has demonstrated good psychometric properties, including support for internal reliability and test–retest reliability, with most subscales showing Cronbach’s alpha values of approximately .65–.70 (34, 52). Cronbach’s alpha for the short version in the present study ranged from .87 at T1 to.90 at T7.

#### 2.4.6 Mystical-type experiences

Mystical-type experiences were measured using the 30-item Mystical Experience Questionnaire (MEQ30) (19, for validation in Scandinavian samples see 53, 54). The MEQ30 was administered at T3. The questionnaire assesses four dimensions of mystical-type phenomena: mystical experiences (e.g., unity, sacredness, noetic quality), transcendence of time and space, ineffability, and positive mood. Items are rated on a 6-point Likert scale ranging from 0 (“Not at all”) to 5 (“Extremely; more than ever”), with higher scores indicating stronger mystical-type experiences. Example item: “Feeling that you experienced eternity or infinity”. Previous research has demonstrated excellent internal reliability for the MEQ30 (α = 0.86–0.97; 19). In the present study, Cronbach’s alpha was .97 for the total scale.

### 2.5 Data collection

Data were collected through psychedelicsurvey.com (55), where participants registered and received automated survey links. Surveys were created in Qualtrics (56) and linked through unique participant codes generated by the platform to ensure consistent longitudinal data while maintaining participant anonymity. The research team did not have access to personal identifying information, IP addresses, or any key enabling re-identification of participants. Participants could withdraw from the study at any time. The study adhered to the Helsinki Declaration (57) and local directives from Stockholm University. The project received an advisory opinion from the Swedish Ethical Review Authority (registry number 2022-03652-01), concluding that formal ethical approval was not required. The study was pre-registered at OSF (43). The seven surveys were administered at the time points shown in **Table 1**, **Figure 1**.

#### 2.5.1 Missing data

The unexpected closure of the survey platform in December 2024 resulted in missing follow-up data for several participants, particularly at the 3-, 6-, and 12-month assessments. 34 participants with baseline data did not meet the longitudinal inclusion criteria for the main analyses, which were based on 173 participants. In addition, the connectedness measure was introduced two months into data collection and was therefore not administered at the early timepoints for the first 30 participants. Missing data (see **Table 1** for dropout at each timepoint) were handled using mixed-effects models estimated with restricted maximum likelihood (REML), allowing all available observations to contribute to the longitudinal analyses under the assumption that data were missing at random.

### 2.6 Statistical analyses

#### 2.6.1 Longitudinal and mediation analyses

To examine changes in mental wellbeing over time (RQ1), linear mixed-effects models were used to estimate change in SWEMWBS at each follow-up timepoint (T2–T7) relative to baseline (T1). Time was treated as a categorical fixed effect, with baseline (T1) as the reference category. Models included random intercepts and adjusted for gender and prior psychedelic experience. Age and psychedelic dose were initially included as covariates but were ultimately omitted, as missing values on these variables would have reduced the analytic sample by 29 participants. Baseline wellbeing (grand-mean centered) was included both as a main effect and in interaction with timepoint, the latter to examine whether wellbeing trajectories differed by baseline level.

To further examine the psychological processes associated with changes in wellbeing (RQ2), exploratory mediation analyses were conducted to test whether changes in connectedness (WCS), neuroticism/trait anxiety, spiritual transcendence (STS), and absorption statistically accounted for observed changes in SWEMWBS over time. Both individual mediation models and a multiple mediation model were estimated. Indirect effects were evaluated using percentile confidence intervals from a cluster bootstrap resampling participants. Each model was fitted at T4–T7 (with T1 as baseline) within the subsample of participants with available data on the relevant mediator.

To examine the role of mystical-type experiences (RQ3), three sets of analyses were conducted. First, additional linear mixed-effects models tested whether mystical-type experiences predicted changes in wellbeing over time, by including an interaction term between MEQ30 scores and timepoint. Second, conditional indirect effects were estimated to assess whether the indirect pathways identified in RQ2 varied as a function of MEQ30 intensity—that is, whether more intense mystical experiences were associated with greater changes in each mediator and, in turn, with wellbeing. Third, in an exploratory analysis, we examined whether any such pathway was specific to connectedness by comparing models in which the MEQ30 × timepoint interaction was adjusted for connectedness versus for the remaining mediators. In the wellbeing outcome models, baseline wellbeing (grand-mean centered) was included both as a main effect and in interaction with timepoint; gender and prior psychedelic experience were included as covariates.

Linear mixed-effects models were estimated using restricted maximum likelihood (REML), which uses all available observations and yields unbiased estimates under a missing-at-random assumption, allowing the inclusion of participants with incomplete repeated-measures data. To assess the tenability of this assumption, attrition analyses examined whether baseline characteristics predicted non-response at follow-up, and a sensitivity analysis tested whether the RQ1 wellbeing trajectory was robust to the inclusion of attrition-related covariates.

All analyses were conducted in R version 4.5.3 (58). Linear mixed-effects models (LMMs) were estimated using the lmer function from the lme4 package (59), with p-values derived via Satterthwaite’s approximation using lmerTest (60), and Wald 95% confidence intervals for fixed effects. Estimated marginal means were obtained using the emmeans package (61), and figures were created using ggplot2 (62).

#### 2.6.2 Comparison to Swedish population

A supplementary analysis compared the proportion of participants classified as having good mental wellbeing with Swedish population estimates reported by the Public Health Agency of Sweden. Good mental wellbeing was defined according to the Agency’s SWEMWBS-based criterion (metric SWEMWBS score > 21, corresponding to a raw score ≥ 24). Analyses were conducted at baseline (T1) and the 3-month follow-up (T5) using all available valid SWEMWBS data. Exact binomial tests were used to compare the observed proportions with the Swedish population benchmark (83.7%). A sensitivity analysis was conducted among participants with valid SWEMWBS data at both T1 and T5 to assess whether findings were influenced by differences in sample composition across timepoints.

## 3 Results

Analyses were based on all individuals who completed the baseline assessment (T1) and at least one follow-up assessment between T3 and T7. This resulted in *N* = 173, except for analyses including connectedness where *N* was 143 (see above). To assess potential attrition bias, logistic regression analyses examined whether baseline levels of mystical experiences (MEQ30), connectedness (WCS), neuroticism (anxiety facet), spiritual transcendence (STS), and absorption predicted attrition at each follow-up assessment (T4–T7), with each predictor tested in a separate univariate model. Lower mystical experiences (MEQ30) predicted higher attrition at T5 (OR = 0.67, 95% CI [0.51, 0.87], *p* = .003), and lower neuroticism (anxiety facet) predicted higher attrition at T5 (OR = 0.78, 95% CI [0.61, 0.98], *p* = .034) and T7 (OR = 0.77, 95% CI [0.61, 0.98], *p* = .035); no other associations were significant. Re-estimating the RQ1 model with baseline neuroticism (anxiety facet) added as a covariate left timepoint estimates virtually unchanged (largest change < 0.001).

Missingness was not completely at random (MCAR), but is plausibly missing at random (MAR) given the variables included in the models. The attrition-related variables are themselves part of the mediation and moderation models, and the RQ1 trajectory was robust to the inclusion of baseline neuroticism (anxiety facet). MEQ30 was not entered into the RQ1 model, as it indexes the retreat experience itself rather than a baseline characteristic.

### 3.1 RQ1 - change in mental wellbeing over time

Linear mixed-effects models indicated significant improvements in mental wellbeing following the retreat, thus supporting hypothesis 1 (see **Table 2**; **Figure 2**). Wellbeing did not differ from baseline at T2, the day before the retreat, but was significantly higher at every timepoint from T3 to T7 (all *p* <.001). The increase was largest immediately after the retreat (T3) and then settled at a descriptively lower but higher-than baseline level from T4 to T7, indicating that gains were largely maintained across the 12-month follow-up. Significant negative interactions between baseline wellbeing and multiple follow-up timepoints suggested that participants with lower initial wellbeing experienced greater improvements over time, whereas participants with higher baseline wellbeing showed smaller changes (**Table 2**). In a sensitivity analysis including age and psychedelic dose as additional covariates (n = 144), neither was significantly associated with wellbeing.

**Table 2:** Fixed effects from a linear mixed model predicting mental wellbeing (SWEMWBS) across time, adjusted for baseline wellbeing, gender, and previous psychedelic experience (N = 872 observations from 173 participants).

Predictor	Est.	SE	95% CI	df	t	p
(Intercept)	3.34	0.05	[3.24, 3.44]	429.4	63.98	<.001
Time point T2	0.06	0.06	[−0.05, 0.16]	700.7	1.02	.307
Time point T3	0.86	0.05	[0.76, 0.97]	698.8	15.94	<.001
Time point T4	0.53	0.06	[0.42, 0.64]	702.4	9.65	<.001
Time point T5	0.51	0.06	[0.39, 0.63]	725.6	8.21	<.001
Time point T6	0.44	0.07	[0.30, 0.57]	740.5	6.26	<.001
Time point T7	0.46	0.08	[0.31, 0.62]	750.5	5.97	<.001
Wellbeing at T1 (centered)	1.02	0.06	[0.91, 1.13]	703.7	17.98	<.001
Gender (male)	0.12	0.05	[0.01, 0.22]	169.4	2.22	.028
Previous experience (Yes)	−0.07	0.05	[−0.17, 0.04]	169.4	−1.22	.224
T2 × Wellbeing at T1 (centered)	−0.29	0.07	[−0.43, −0.15]	703.7	−3.93	<.001
T3 × Wellbeing at T1 (centered)	−0.75	0.07	[−0.90, −0.61]	701.7	−10.33	<.001
T4 × Wellbeing at T1 (centered)	−0.58	0.07	[−0.72, −0.44]	701.5	−7.93	<.001
T5 × Wellbeing at T1 (centered)	−0.52	0.08	[−0.68, −0.36]	721.0	−6.46	<.001
T6 × Wellbeing at T1 (centered)	−0.55	0.09	[−0.72, −0.38]	733.8	−6.25	<.001
T7 × Wellbeing at T1 (centered)	−0.65	0.10	[−0.85, −0.45]	745.5	−6.43	<.001

Timepoint T1 served as the reference category. Baseline wellbeing was grand mean-centered.

Results are based on a linear mixed-effects model predicting mental wellbeing across follow-up timepoints, controlling for baseline wellbeing, gender, and prior psychedelic experience. T1 served as the reference category. Positive coefficients indicate higher wellbeing relative to baseline (T1). Baseline wellbeing was mean-centered. Estimates are fixed-effect regression coefficients, and 95% confidence intervals represent Wald confidence intervals for the fixed effects. Models were estimated using maximum likelihood with random intercepts for participants, allowing inclusion of participants with incomplete repeated-measures data under missing-at-random assumptions.

**Figure 2:**
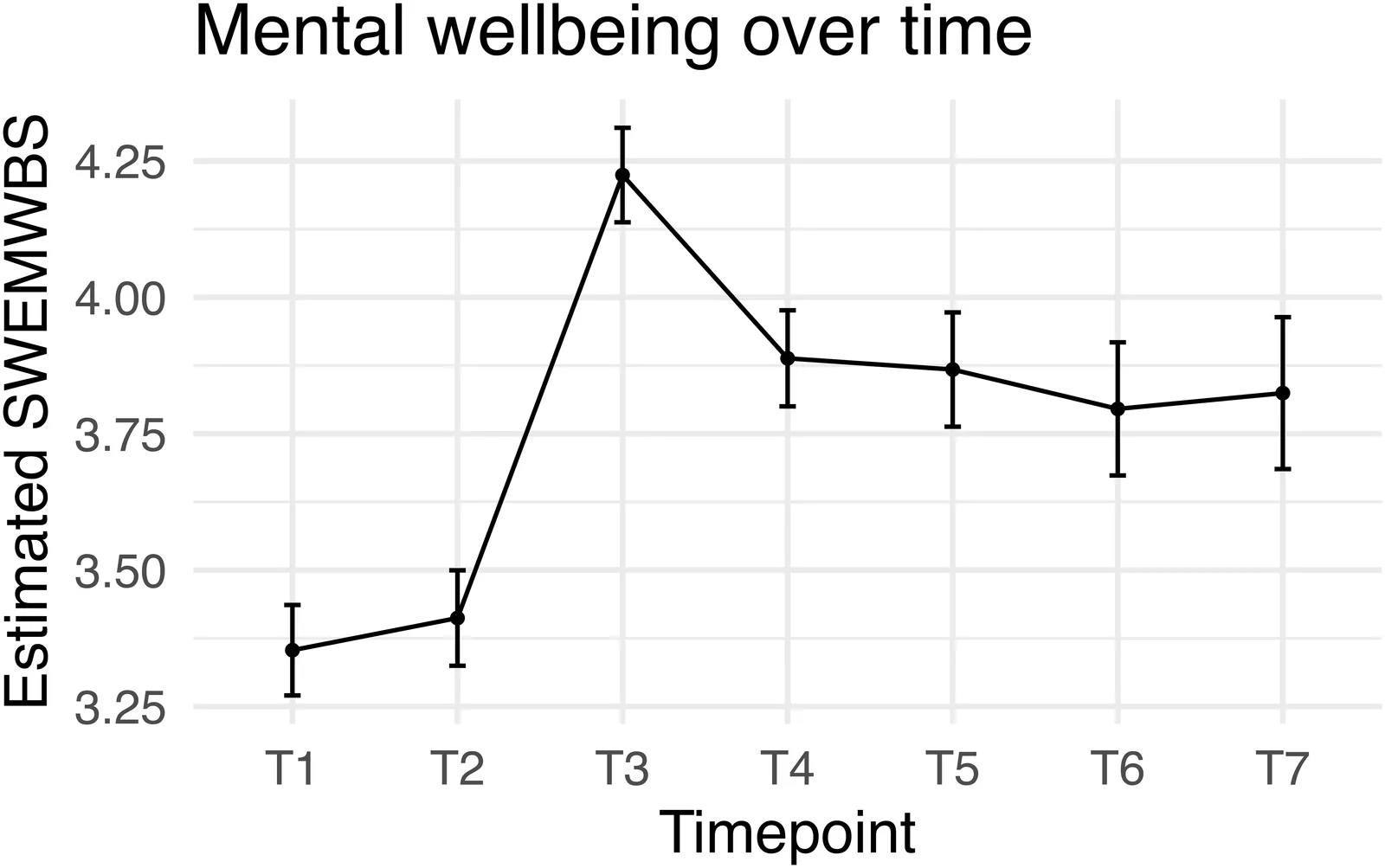
Mental wellbeing (SWEMWBS) across timepoints. Points represent model-estimated SWEMWBS means from a linear mixed-effects model with random intercepts for participants, adjusted for baseline wellbeing, gender and previous psychedelic experience. Error bars represent 95% confidence intervals around the estimated marginal means. Assessments were conducted two weeks before the retreat (T1) and the day before the retreat (T2). The retreat took place between T2 and T3. Follow-up assessments were conducted the day after the retreat (T3), and at two weeks (T4), three months (T5), six months (T6), and twelve months (T7) after the retreat.

### 3.2 RQ2 - mediation of wellbeing changes

To examine how changes in potential mediators were associated with improvements in mental wellbeing, we first conducted single-mediator path analyses for each proposed mediator, followed by a multiple mediation model including all variables simultaneously. All models were estimated using linear mixed-effects models (LMMs).

In the single-mediator models, all proposed mediators showed significant indirect effects on wellbeing (**Table 3**). Increases in connectedness (WCS) showed the strongest mediation effect (*ab* = 0.321, 62.4%, *p* <.001), followed by reductions in neuroticism (*ab* = 0.207, 41.4%, *p* <.001). Importantly, increases in spiritual transcendence (STS) were also significantly associated with improvements in wellbeing in the single-mediator model (*ab* = 0.171, 35.9%, *p* <.001), as were increases in absorption (*ab* = 0.104, 21.1%, *p* <.001), indicating that all four variables independently contributed to wellbeing change when considered in isolation.

**Table 3:** Single-mediator models of change in wellbeing.

Mediator	a-path	b-path	Indirect effect (ab)	95% CI	% mediated	c′-path
WCS	13.55***	0.024***	0.321	[0.236, 0.413]	62.4%	0.186*
Neuroticism	−0.727***	−0.285***	0.207	[0.153, 0.264]	41.4%	0.290***
STS	0.380***	0.448***	0.171	[0.119, 0.227]	35.9%	0.317***
Absorption	0.373**	0.279***	0.104	[0.059, 0.160]	21.1%	0.391***

All indirect effects significant based on bootstrap confidence intervals.

a-path, average effect of timepoint (T4–T7) on mediator.

b-path, effect of mediator on wellbeing controlling for time.

c′-path, remaining direct effect of time after inclusion of mediator.

Indirect effects (ab) estimated using cluster bootstrap confidence intervals with participant-level resampling. *p <.05, **p <.01, ***p <.001.

In the multiple mediation model, where shared variance between mediators was accounted for, connectedness (*ab* = 0.243, 46.8%), neuroticism/trait anxiety (*ab* = 0.111, 21.3%), and absorption (*ab* = 0.049, 9.4%) retained significant unique indirect effects, each with bootstrap confidence intervals excluding zero. The indirect effect of STS was no longer significant (*ab* = −0.025, 95% CI [−0.087, 0.028]), suggesting that its association with wellbeing was explained by overlap with the other mediators in the model — in particular connectedness and absorption, with which STS was substantially correlated across follow-up timepoints (*r* = .56–.72 and .58–.68, respectively) (**Figure 3**; **Table 4**). Overall, the combined indirect effect accounted for a substantial proportion of the total effect of time on wellbeing (*ab* = 0.377 of total effect = 0.519), corresponding to approximately 72.7% mediated.

**Table 4:** Joint Multiple mediation model predicting wellbeing.

Mediator	a-path	b-path	p	Indirect effect (ab)	95% CI	% mediated
WCS	13.553	0.018	<.001	0.243*	[0.154, 0.353]	46.8%
Neuroticism	−0.733	−0.151	<.001	0.111*	[0.057, 0.170]	21.3%
STS	0.380	−0.065	.231	−0.025	[−0.087, 0.028]	−4.8%
Absorption	0.379	0.129	<.001	0.049*	[0.015, 0.090]	9.4%

Bootstrap confidence intervals showed that connectedness, neuroticism (anxiety facet), and absorption showed significant indirect effects (95% CI excluding zero) in the joint model, which included all four mediators simultaneously. Indirect effects represent unique mediation effects controlling for shared variance among mediators. Confidence intervals excluding zero were interpreted as statistically significant. Significant effects are indicated in bold and marked with *(*p* <.05).

**Figure 3:**
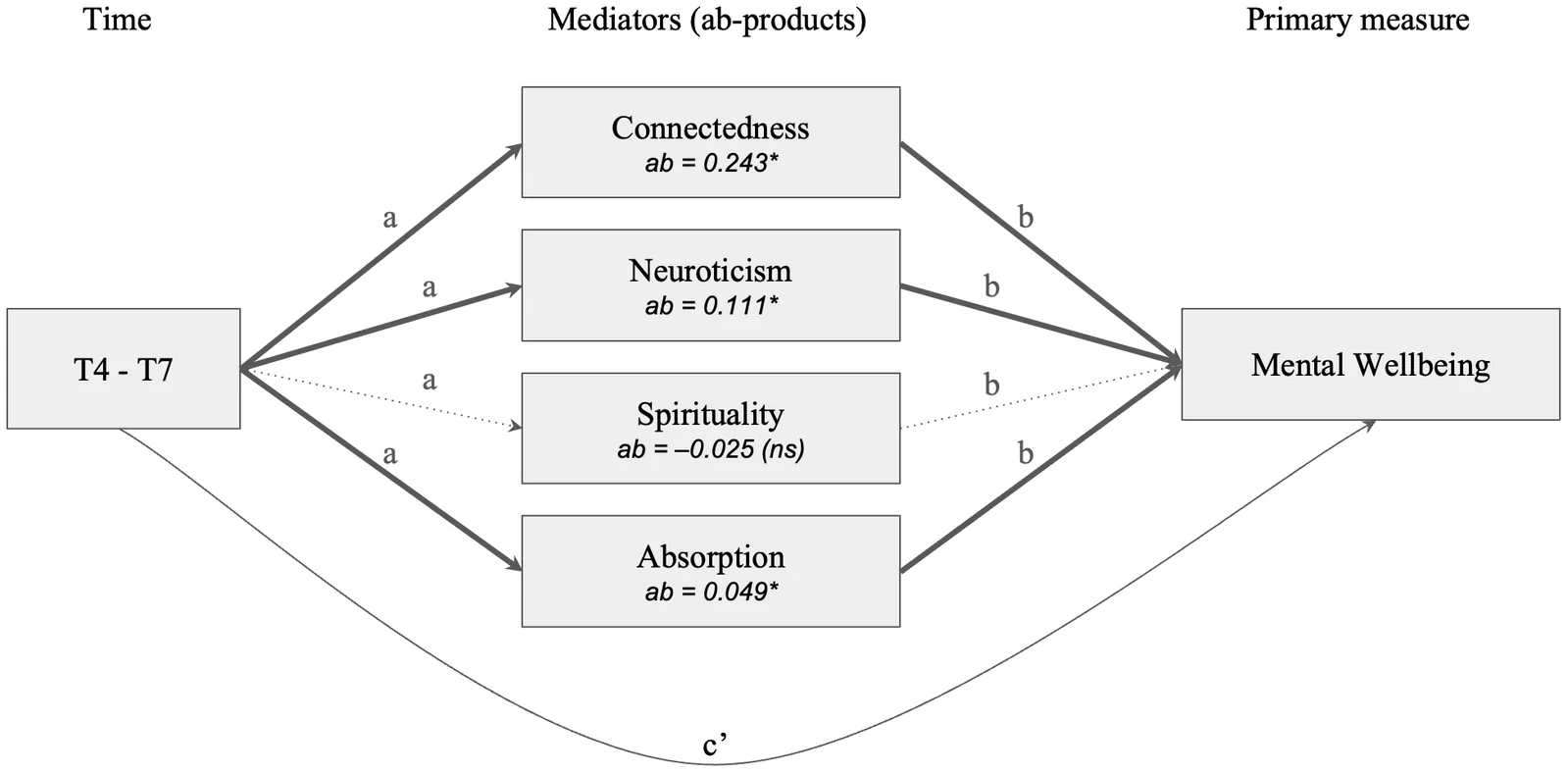
Multiple mediation model of the association between time since retreat and mental wellbeing. The model shows indirect associations through connectedness, neuroticism (anxiety facet), spirituality, and absorption. Indirect paths are represented by ab products, where a-paths show how time affects each mediator; b-paths show associations between changes and mental wellbeing; and c′ represents the direct association of time after accounting for all mediators. Across follow-up assessments (T4–T7), the mediators collectively accounted for approximately 69.5%–77.7% of the total association between time and mental wellbeing. *Indicates significant association. (ns) indicates not significant.

### 3.3 RQ3 mystical-type experiences as a predictor of psychological change and wellbeing (MEQ30)

To examine whether mystical-type experiences (MEQ30) predicted changes in wellbeing over time (RQ3), a linear mixed-effects model was estimated including an interaction between MEQ30 and timepoint (**Figure 4**). MEQ30, assessed the day after dosing (T3), was treated as a predictor of subsequent psychological change and wellbeing. The MEQ30 × timepoint interactions, reported below, tested whether MEQ30 predicted the magnitude of change from baseline at each timepoint.

**Figure 4:**
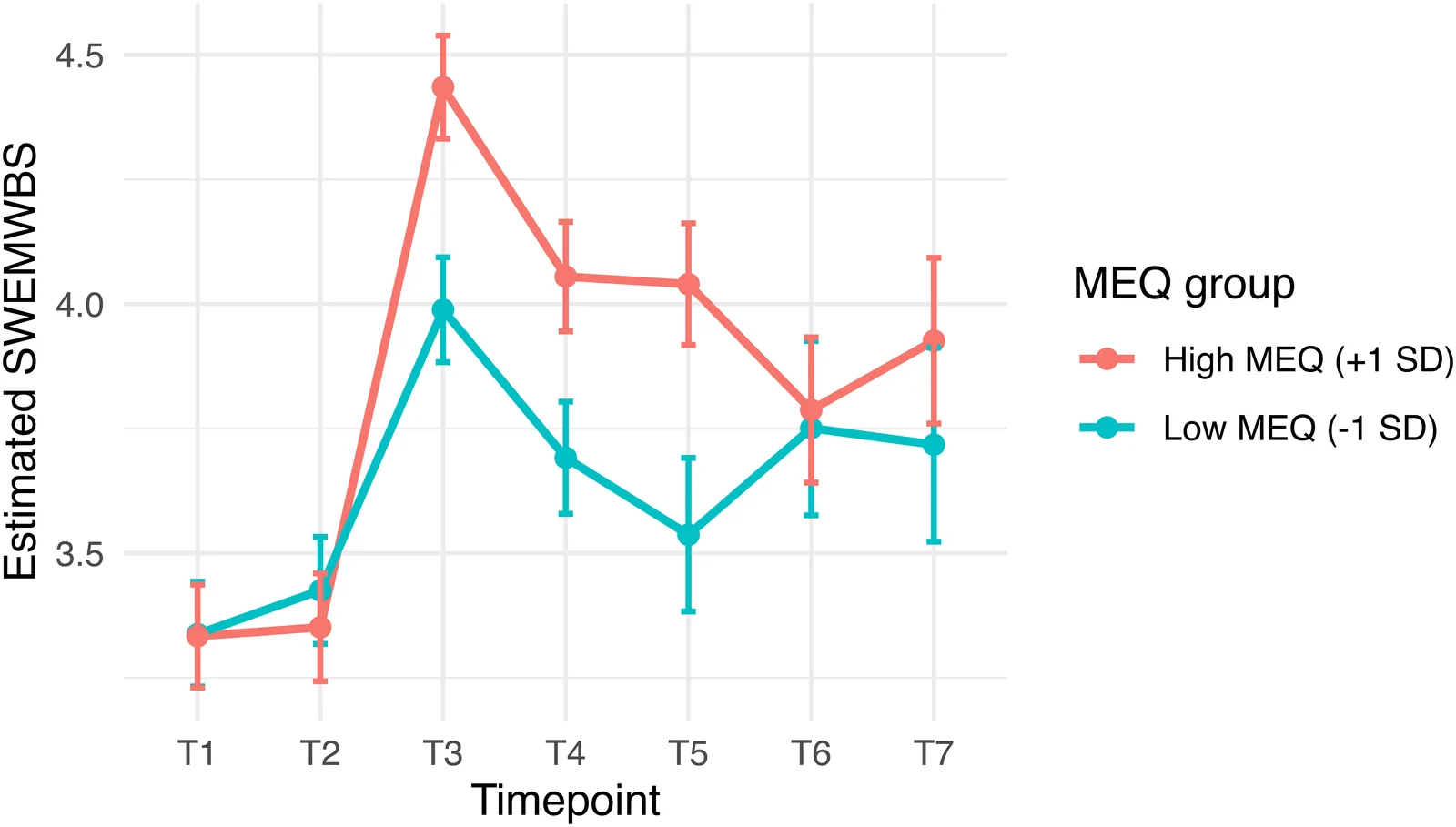
Changes in mental wellbeing by intensity in mystical-type experiences. Predicted values of mental wellbeing (SWEMWBS) across timepoints for individuals reporting low (−1 SD) and high (+1 SD) levels of mystical-type experiences (MEQ30). Estimates are derived from a linear mixed-effects model including timepoint, baseline wellbeing, and their interaction with MEQ30, controlling for gender and prior psychedelic experience. Error bars represent 95% confidence intervals.

Higher MEQ30 scores were associated with greater wellbeing at T3 (0.225, SE = 0.044, *p* <.001), T4 (0.184, SE = 0.046, *p* <.001), and T5 (0.253, SE = 0.055, *p* <.001), whereas the interaction effects at later follow-ups were weaker or non-significant (see **Figure 4**). Hence, hypothesis 2 was partly supported. Exploratory analyses further examined whether the association between MEQ30 and wellbeing improvements was statistically mediated by changes in the proposed mediators. Firstly, separate linear mixed-effects models indicated that higher MEQ30 scores were associated with greater increases in connectedness (WCS), spiritual transcendence (STS), and absorption, as well as greater reductions in neuroticism (trait anxiety) across several follow-up timepoints (see **Table 5**). Secondly, individual bootstrap analyses of conditional indirect effects for the mediators showed that each statistically mediated the pathway between MEQ30 and improvements in wellbeing at several follow-up timepoints, although the strongest effects overall were seen for connectedness.

**Table 5:** Mystical-type experiences (MEQ30) predicting psychological changes and wellbeing improvements.

Mediator	Time	MEQ30 × mediator	SE	p	Conditional indirect effect (ab)	95% CI
WCS	T4	5.478	1.237	<.001	0.130	[0.074, 0.196]
	T5	4.985	1.430	<.001	0.118	[0.037, 0.212]
	T6	2.103	1.749	.230	0.050	[−0.031, 0.135]
	T7	3.380	2.034	.098	0.080	[0.001, 0.187]
Neuroticism	T4	−0.153	0.073	.037	0.044	[0.009, 0.086]
	T5	−0.197	0.087	.024	0.056	[0.016, 0.106]
	T6	−0.167	0.096	.082	0.048	[−0.003, 0.108]
	T7	−0.373	0.105	<.001	0.106	[0.045, 0.177]
STS	T4	0.208	0.037	<.001	0.093	[0.046, 0.152]
	T5	0.183	0.044	<.001	0.082	[0.036, 0.137]
	T6	0.099	0.049	.045	0.044	[−0.011, 0.112]
	T7	0.148	0.054	.006	0.066	[−0.015, 0.181]
Absorption	T4	0.213	0.058	<.001	0.059	[0.028, 0.101]
	T5	0.296	0.070	<.001	0.082	[0.028, 0.145]
	T6	0.051	0.077	.507	0.014	[−0.040, 0.078]
	T7	0.214	0.084	.011	0.060	[0.000, 0.135]

Conditional indirect effects (ab) were estimated using cluster bootstrap confidence intervals (1,000 resamples). Significant indirect effects are indicated where the 95% confidence interval excluded zero. MEQ30, Mystical Experience Questionnaire; WCS, Watts Connectedness Scale; STS, Spiritual Transcendence Scale.

Finally, additional exploratory models examined whether this indirect effect was specific to connectedness relative to the other mediators. The MEQ30 × timepoint interaction, significant at T4 (0.185, *p* <.001) and T5 (0.255, *p* <.001) without mediators, was substantially reduced and no longer significant when connectedness was included (T4: 0.010, *p* = .841; T5: 0.100, *p* = .064). In contrast, the interaction remained significant at these timepoints when controlling for the other mediators without connectedness (T4: 0.086, *p* = .042; T5: 0.140, *p* = .005), suggesting that connectedness may represent a unique pathway through which mystical-type experiences are associated with improvements in wellbeing.

### 3.4 Comparison to the Swedish population

To contextualize the wellbeing levels of retreat participants from a public health perspective, baseline wellbeing was compared with Swedish population benchmarks. At baseline, 97 of 189 participants with valid SWEMWBS data were classified as having good mental wellbeing, according to the Public Health Agency of Sweden’s definition, corresponding to 51.3%. This was substantially lower than the 2024 Swedish population estimate of 83.7% (exact binomial test, *p* <.001). At T5, the proportion had increased to 82 of 109 participants, corresponding to 75.2%, indicating that the retreat sample had moved closer to the Swedish population benchmark, although it remained descriptively lower (*p* = .011). In the sensitivity analysis restricted to the 104 participants with valid SWEMWBS data at both T1 and T5, a similar pattern was observed: the proportion with good mental wellbeing increased from 48.1% at T1 to 74.0% at T5. This suggests that the observed improvement was not solely due to differences in sample composition across timepoints.

## 4 Discussion

This naturalistic study examined changes in mental wellbeing following participation in a guided psilocybin retreat from a public mental health perspective. Overall, participants reported substantial and largely sustained improvements in wellbeing across the 12-month follow-up period. Among the psychological variables examined, changes in connectedness emerged as the strongest explanatory pathway, though reductions in the anxiety facet of neuroticism and absorption also contributed uniquely to improved wellbeing in a joint model. In addition, more intense mystical-type experiences during the retreat were associated with greater subsequent improvements in wellbeing up to three months post the retreat. This link appeared to be carried largely by connectedness: more intense mystical experiences predicted greater increases in connectedness, which in turn was the change most consistently associated with sustained wellbeing. Together, the findings suggest that especially post-retreat changes in connectedness, and the intensity of mystical experiences during the acute phase, may represent important psychological pathways associated with long-term wellbeing following participation in a guided psilocybin retreat.

### 4.1 Discussion of key findings

The present study found significant improvements in mental wellbeing following participation in a psychologist-guided psilocybin retreat. Improvements were evident immediately after the retreat and remained elevated throughout the 12-month follow-up period. Also, participants with lower baseline wellbeing showed the greatest improvements (though see below for a caveat).

Among all investigated candidate psychological mediators, connectedness emerged as the most robust. While all four presumed mediators were associated with wellbeing when examined individually, only connectedness made a substantial contribution, though neuroticism (trait anxiety) and absorption also retained significant unique indirect effects in the multiple mediation model. Thus, connectedness accounted for the largest share of the mediated effect. These findings are consistent with previous research identifying connectedness as a central predictor of psychological wellbeing and positive mental health (4, 16, 18). Alongside connectedness, reductions in neuroticism (trait anxiety) uniquely contributed to improvements in wellbeing, suggesting that increased emotional stability may represent a second important pathway of change. Reductions in neuroticism have previously been linked to improved wellbeing, and may reflect enduring psychological changes following significant life experiences (31, 33). Absorption likewise retained a small but significant unique contribution, consistent with its proposed role in facilitating openness and engagement with altered states of consciousness (35, 36). Its modest independent effect suggests that the capacity for deep experiential engagement may support wellbeing change beyond what is captured by connectedness and emotional stability.

Spiritual transcendence was associated with wellbeing improvements when examined separately, but its effect was no longer significant once overlap with the other mediators were taken into account. Consistent with this, spiritual transcendence was remarkably strongly correlated with both connectedness (*r* = .56–.72) and absorption (*r* = .58–.68) across follow-up timepoints, suggesting that it may capture psychological processes largely shared with these constructs rather than contributing independently to wellbeing change. Indeed, our findings may be taken to suggest that, from a psychological point of view, spirituality largely amounts to connectedness plus absorption.

The present study obviously cannot disentangle the effects of psilocybin from those of the broader retreat environment, thus the relative contribution of pharmacological and contextual factors remains unclear. Nevertheless, the prominence of connectedness raises the possibility that elements of the retreat context, including shared preparation, group processes, and collective integration, may have contributed to the observed increases in connectedness and, indirectly, to improvements in wellbeing.

Mystical-type experiences were also associated with greater improvements in wellbeing, particularly during the earlier follow-up period, up to three months post the retreat. Higher MEQ30 scores predicted larger increases in connectedness, spirituality, and absorption, as well as greater reductions in the anxiety facet of neuroticism. Connectedness again provided the most consistent indirect pathway, linking the mystical experience to wellbeing. Indeed, the association between mystical experience and wellbeing change was substantially attenuated and no longer significant once connectedness was included in the model. These findings suggest a pathway in which psychedelically induced mystical experiences lead to an increased sense of connectedness with oneself, others and, one’s surroundings, which in turn may promote greater wellbeing. This is consistent with previous suggestions regarding the centrality of both mystical experiences (e.g., 40) and connectedness (e.g., 26, 27) to the positive effects of psychedelics. The weaker associations between mystical-type experiences and change in wellbeing at later follow-ups may further indicate that such experiences need to be translated into more enduring psychological changes, such as increased connectedness, to sustain wellbeing over time.

Notably, retreat participants reported substantially lower wellbeing than the Swedish population at baseline. From a public mental health perspective, the findings are noteworthy because they concern participants attending a retreat outside a conventional clinical treatment setting, and assess wellbeing rather than clinical symptom outcomes. However, participants may have entered the retreat with a range of motivations, including the possibility of seeking relief from psychological difficulties, and the present study did not assess participants’ reasons for attending or their primary treatment goals. Participants underwent medical and psychiatric screening for conditions and risk factors associated with elevated risk from psilocybin administration, including bipolar disorder and a family history of psychosis. However, depression and anxiety were not exclusion criteria for participation, and psychiatric diagnoses were not systematically assessed or reported in the present study. Consequently, the sample should not be assumed to be free from psychiatric or subclinical psychological difficulties, which may have contributed to the relatively low baseline wellbeing. The extent to which the findings generalize to psychologically healthy populations or to individuals seeking symptom reduction therefore remains unclear. The results suggest that connectedness may represent an important correlate of mental wellbeing not only in clinical contexts but also in broader populations (but see below).

Our findings should be interpreted alongside recent critical perspectives within psychedelic research. Several authors have argued that positive findings attributed to psychedelics may be amplified by methodological problems (discussed in the next section) publication bias, expectancy effects, selective reporting, and methodological inconsistencies (63–65). Moreover, qualitative reports describe potential risks including psychological destabilization, dependency, shame, difficulties integrating experiences, and problematic power dynamics in psychedelic settings (64). The present findings therefore should not be interpreted as evidence that psychedelic retreats are universally beneficial. Rather, they suggest that positive outcomes may occur within carefully structured settings that include screening, psychological preparation, support during dosing, and post-retreat integration. This may be particularly important given that just over half of the participants (56.6%) were psychedelic-naïve, suggesting that guided retreats may constitute a first point of contact with psychedelics for many individuals. Notably, prior psychedelic experience was not significantly associated with wellbeing, suggesting that the observed improvements were not limited to those with previous experience.

### 4.2 Methodological considerations

When interpreting our findings, several methodological limitations should be considered. First, the absence of a control group and blinding means that expectancy effects cannot be ruled out. Participants self-selected into the retreat, paid for participation, and may therefore have entered the study with strong expectations regarding personal growth and wellbeing. In addition, unmeasured factors such as concurrent therapy, medication changes, or major life events may have influenced outcomes during the 12-month follow-up period. Further, that the participants with lower baseline wellbeing showed the greatest improvements may be explained by regression to the mean (66). Although the sustained improvements across multiple follow-up assessments suggest this is unlikely to fully account for the findings, its potential contribution cannot be ruled out in the absence of a control group. While expectancy effects may have contributed to the magnitude of the observed improvements, the mediation patterns observed in the present study are broadly consistent with previous psychedelic research highlighting connectedness, reductions in neuroticism (anxiety facet), and mystical-type experiences as potential pathways underlying positive psychological outcomes (16–18, 27, 42, 67). Also, these limitations are not unique to naturalistic studies. Supposedly placebo-controlled clinical psychedelic trials frequently face difficulties maintaining blinding, as the subjective effects of psychedelics often reveal treatment allocation and may influence outcomes through expectancy effects (68). Expectancy therefore remains a broader methodological challenge across the field and should be assessed more directly in future research. Note, however, a recent study failed to see larger effects due to expectancy in a psychedelic therapy trial, suggesting that the possible limitations of expectancy effects may be exaggerated (69).

Second, attrition and missing data should also be considered. Follow-up completion declined over time, partly due to the unexpected closure of the survey platform in late 2024, which prevented data collection from several participants. Attrition analyses indicated some selective dropout, with lower mystical experiences and lower neuroticism (trait anxiety) associated with non-response at certain follow-up assessments. However, re-estimating the primary wellbeing model with baseline neuroticism included produced virtually identical timepoint estimates, suggesting that attrition was unlikely to materially influence the observed wellbeing trajectory.

Third, the analytic approach had inherent limitations. Although mediation analyses are commonly used to explore mechanisms of change, causal interpretations are limited in the absence of a control group. In addition, mediators and wellbeing outcomes were assessed at the same follow-up timepoints, preventing firm conclusions regarding temporal precedence between the proposed mediators and changes in wellbeing. The mediation findings should therefore be considered hypothesis-generating rather than definitive. Furthermore, all outcomes were assessed through self-report measures, introducing potential biases related to shared method variance, expectancy effects, and limited self-insight. Relatedly, the MEQ30 captures mystical-type experiences specifically, and the relative contribution of other acute experiences to long-term wellbeing remains an open question (70).

Finally, the characteristics of the sample limit generalizability. Participants reported substantially lower wellbeing than the Swedish population at baseline. This likely reflects, at least in part, self-selection, whereby individuals seeking personal development or transformative experiences may differ systematically from the general population. However, the finding may also indicate that substantial variation in wellbeing exists within ostensibly healthy populations and that individuals experiencing lower wellbeing may be particularly motivated to seek interventions aimed at personal growth and psychological flourishing.

Despite these limitations, the present study had several strengths. All participants attended the same retreat model, reducing variability in intervention delivery. The naturalistic design enhances ecological validity by examining outcomes in a real-world setting rather than a laboratory environment. The relatively large sample and 12-month follow-up period provide rare insight into longer-term wellbeing trajectories following a psilocybin retreat. Moreover, by focusing on positive mental wellbeing rather than symptom reduction alone, the study contributes to the growing literature examining psychedelics from a public mental health perspective.

### 4.3 Ethical and legal considerations

The aim of the present study was not to evaluate the ethical or legal status of psychedelic retreats, nor to advocate their use, but to examine psychological processes associated with wellbeing in a real-world retreat context. Consequently, the findings should be interpreted as evidence of associations observed within a specific retreat model rather than as support for psychedelic retreats more generally. Participants were volunteers attending a psychologist-guided retreat, and the observed associations may not generalize to clinical populations or to other psychedelic settings. As researchers, we also stress our responsibility to avoid overstating benefits or inadvertently legitimizing practices that may carry legal or psychological risks.

From a public mental health perspective, questions of accessibility and equity are also important to consider. Participation required substantial financial resources and international travel, limiting access primarily to socioeconomically advantaged individuals. Consequently, even if such interventions prove beneficial, their broader contribution to population mental health will depend on issues of accessibility, regulation, participation safety, and equitable implementation.

Prior to study initiation, the research protocol was submitted to the Swedish Ethical Review Authority (Etikprövningsmyndigheten; application no. 2022-03652-01) for assessment. Following review, the Authority concluded that the study did not fall within the scope of the Swedish Ethical Review Act and therefore did not require formal ethical approval. Specifically, the Authority determined that the study did not involve any research intervention as defined by the Act, nor did it involve the processing of personal data in a manner covered by the Act. Consequently, the application was dismissed from formal ethical review, and an advisory opinion was issued.

The study was a naturalistic, observational survey study that did not influence participants’ decisions to attend the retreat or any aspect of the retreat program. Participants had independently enrolled in the retreat prior to recruitment. The research consisted solely of anonymous online questionnaires administered before and after the retreat. No identifiable personal data were collected or processed by the research team, participation was entirely voluntary, and all participants provided informed consent electronically before enrolment.

## 5 Conclusions and future directions

Our results highlight connectedness as a potentially important process in understanding long-term wellbeing following psychedelic experiences. Given the growing recognition of loneliness and social disconnection as major public health concerns, the prominence of connectedness in the present study suggests that social and relational dimensions may warrant greater attention within psychedelic research and practices. Future studies should investigate whether the associations observed here can be replicated in controlled designs and whether group-based preparation and integration contribute to wellbeing outcomes. Comparative studies examining the relative influence of psychedelic, social, and contextual factors would be particularly valuable.

Future research would also benefit from complementing self-report measures with more objective indicators of functioning and wellbeing, such as healthcare utilization, sickness absence, occupational functioning, or observer-rated outcomes. Such approaches may help clarify the extent to which reported improvements translate into meaningful changes in everyday life.

In closing, this study contributes to the growing naturalistic psychedelic literature by providing one of the longer follow-ups of a psychologist-guided psilocybin retreat model in a non-clinical retreat setting. Participation in the retreat was associated with sustained improvements in mental wellbeing over 12 months. Mystical-experience facilitated increases in connectedness emerged as the strongest mediating pathway, followed by neuroticism (anxiety facet) and absorption. Together, these findings highlight the potential importance of both social and intrapersonal processes in understanding how psychedelic experiences may be associated with long-term wellbeing.

## Data Availability

The raw data supporting the conclusions of this article will be made available by the authors, without undue reservation.
